# Supplementary material for: Temperature dependence of adsorption hysteresis in flexible metal organic frameworks
Source: Commun Chem. 2020 Dec 10;3:186. doi: 10.1038/s42004-020-00429-3 (PMC9814463; doi:10.1038/s42004-020-00429-3)
Supplement: Supplementary file 1 — Supplementary Information [file 42004_2020_429_MOESM1_ESM.docx]

**SUPPLEMENTARY INFORMATION**

**Temperature Dependence of Adsorption Hysteresis in Flexible Metal Organic Frameworks**

Shamsur Rahman^1^, Arash Arami-Niya^1, 2^, Xiaoxian Yang^1^, Gongkui Xiao^1^, Gang (Kevin) Li^1,3^, Eric F. May^1,^*

^1^ Fluid Science & Resources Division, School of Engineering, University of Western Australia, Crawley, WA 6009, Australia

^2^ Discipline of Chemical Engineering, Western Australian School of Mines: Minerals, Energy and Chemical Engineering, Curtin University, GPO Box U1987, Perth, WA 6845, Australia

^3^ Department of Chemical Engineering, The University of Melbourne, Melbourne, Victoria 3010, Australia

*E-mail: [eric.may@uwa.edu.au](mailto:eric.may@uwa.edu.au) ORCID: 0000-0001-5472-6921

## Supplementary Note 1. Derivation of the LJMY model for stepped sorption isotherms.

The equation for *q*_LJMY_ is based on a conceptual model, which assumes that the increased capacity associated with the step is a result of a localized structural phase transition in the adsorbent material (MOF), triggered by the presence of an adsorbed phase. While the model may be applied to regress hysteretic stepped isotherms for either so-called breathing or gating MOFs, the conceptual description detailed below applies to gating MOFs, where in the absence of an adsorbate the crystal is in a narrow-pore (np) state ^1^. Before the coverage of the adsorbed phase is sufficient, the interfacial energy penalty associated with the MOF surface forces the crystal to minimize its area.^2^ However, as the amount of adsorbed phase in a local region of the surface increases with gas pressure, the interfacial free energy of the MOF in that local region decreases. At some threshold value, the reduction in the interfacial free energy is sufficient to drive a localized crystalline phase transition of the MOF, with the resulting structure having a much larger surface area: this may be called the large-pore (lp) state.^2^ The spatial extent of the crystalline transition is limited by the amount of adsorbed phase available to lower the interfacial energy of the MOF surface. Further increases in gas phase pressure and adsorption allow the MOF crystal transition to propagate further throughout the material until the sample reaches its maximum possible surface area.

The conceptual model also assumes that the threshold gas pressure required to induce structural transition in the MOF varies throughout the sample and is described by a Gaussian distribution with a mean and standard deviation of *p*_tr_ and σ, respectively. One possible explanation for this relates to the distribution of pore length scales within the np state. These varying length scales influence the gas pressure, *p*_θ_, required locally for an amount of adsorption sufficient to trigger the structural transition. Pores in the sample with lower threshold pressures trigger a local np to lp transition earlier along the adsorption isotherm, and the variation between pores is the reason that the entire sample does not undergo the transition at a single pressure. This Gaussian distribution would essentially describe the probability density function, Pr(*p*_θ_), for MOF pores in the np state to have undergone the structural transition.

$Pr\left( p_{\theta} \right)=\frac{1}{\sigma\sqrt{2\pi}}\exp\left[ -\frac{\left( p_{\theta}-p_{tr} \right)^{2}}{2 \sigma^{2}} \right]$ ()

The total adsorbed capacity at a given pressure associated with the MOF structural transition is determined by integrating this probability density function

$q_{\mathrm{LJMY}}=Q_{\mathrm{step}}\int_{0}^{p} Pr\left( p_{\theta} \right)dp_{\theta}=\frac{Q_{\mathrm{step}}}{2}\left[ 1+erf\left( \frac{p-p_{tr}}{\sqrt{2}\sigma} \right) \right]$ ()

where *Q*_step_ is the maximum sorption capacity increase resulting from the MOF structural change. To describe a branch of a hysteretic stepped sorption isotherm for a given MOF, the LJMY model may be combined with any classical sorption isotherm model (e.g. Sips, Toth) as required to best describe the data. Here we chose the Langmuir function to minimise the number of adjustable parameters being regressed: the combined function shown in Supplementary eq. (1a) of the main text is therefore the LJMY-Langmuir model.

To regress both branches of a hysteretic isotherm simultaneously a dummy independent variable, *y*, may be introduced into the fit function. During the regression, the value of *y* determines along which branch of the isotherm the data were measured. It can be convenient to set *y* = 0 as corresponding to the desorption branch and *y* = 1 as corresponding to the adsorption branch. This allows quantities that take on different values for the two branches, (e.g. $p_{tr}^{(ads)}$and $p_{tr}^{(des)}$) and must therefore be represented by two separate parameters, to be expressed in terms of *y*. For example, to simultaneously regress Supplementary eq (1) to both branches of a stepped sorption isotherm while constraining four of the parameters (σ, *Q*_step_, *K* and *Q*_m_) to have the same value on each branch, the capacity function can be defined

$q\left( p,y \right)=\frac{Q_{\mathrm{step}}}{2}\left[ 1+erf\left( \frac{p-(1+y \gamma)p_{tr}}{\sqrt{2}\sigma} \right) \right]+Q_{m}\frac{K p}{1+K p}$ ()

where the additional adjustable parameter, *γ*, has been introduced. This allows the transition pressures along the desorption and adsorption branches to be identified as:

$p_{tr}^{(des)}=p_{tr}$ (a)

$p_{tr}^{(ads)}=p_{tr}\left( 1+\gamma\right)$ (4b)

Fundamental simulations capable of modelling responsive adsorption processes in flexible porous materials have been developed ^3^, which allow for a strained framework to exist during the transition between the np and lp phases as the unit cell parameters change. ^4, 5^ The parameters extracted by fitting the LJMY isotherm to experimental data could in principle be related to the predictions of fundamental models that couple pore width with DFT calculations and can account for the effect of strain in the crystal. This might provide a pathway to efficiently quantifying the effect of strain on observed isotherms.

Of the parameters in the LJMY model, the transition width, σ, is the most likely to be influenced by the effects of strain, particularly in those cases where there is hysteresis in its value between the adsorption and desorption branch. For example differences in strain caused by the np to lp transition from those caused by the lp to np transition might manifest themselves in a non-zero δσ = *σ* ^(ads)^ - *σ* ^(des)^ as observed for Co(bdp).

However, the LJMY model does not make any direct reference to crystal strain. Any connection between the LJMY parameters extracted by fitting to sorption isotherm data and more fundamental simulations that do include crystal strain will need to be inferred by analyzing the predictions of those simulations. Additionally, the LJMY model can not treat stepped isotherms where the observed sorption capacity does not increase monotonically with pressure, for example as observed for materials that exhibit negative gas adsorption ^4^. The primary purpose of the LJMY model is to serve as a useful tool for analyzing experimental sorption isotherms measured for particular combinations of guests and flexible adsorbents, and representing those isotherms in simulations of pressure swing adsorption processes based on those materials.

## Supplementary Note 2. Results of LJMY-Langmuir model regression to MOF isotherm data.

Supplementary Tables 1 to 3 show the best-fit parameters obtained from regressing the LJMY-Langmuir model to the MOF isotherm data. For each isotherm, three sets of parameters are listed: a fit to each branch separately and the results of fitting both branches simultaneously with Supplementary eq (3).

Fitting both branches simultaneously with Supplementary eq (3) constrains σ, *Q*_step_, *Q*_m_ and *K* to have the same value in adsorption and desorption, preventing any investigation of hysteresis in these quantities. Particularly for the Fe(bdp) and Co(bdp) data, different values of *Q*_step_ on the two isotherm branches are needed to accurately represent the measured data across all conditions, especially when the hysteresis in transition pressure becomes at higher temperatures. Appreciably worse fits are achieved in these cases if the branches are fit simulatenously.

A possible physical explanation for the hysteresis in *Q*_step_ suggested by fitting the branches separately is that at higher temperatures the properties of the adsorbed phase do not lower the MOF’s interfacial free energy as effectively and hence significantly more adsorption is necessary to induce the np to lp structural transition.

However, care should be taken not to over-interpret the best-fit value of *Q*_step_ obtained from a fit to a single isotherm branch, because it is correlated with *Q*_m_ and *K*. In contrast, *p*_tr_ and σ, which are of primary interest in this work, are robustly orthogonal to the other parameters in the model; their values and uncertainties do not change significantly if the branches are fit simultaneously, although their statistical uncertainties inherently increase if the fit quality deteriorates. Accordingly to more accurately investigate the dependence of *p*_tr_ and σ on temperature and isotherm branch, the values obtained by fitting the independent branches were used in the main text.

Additionally, the potential for correlation between *Q*_step_, *Q*_m_ and *K* means that values of the latter two Langmuir parameters should be interpreted with caution. This is particularly important for fits where there are a relatively small number of data points measured at pressures below the structural transition, and/or where the equilibrium capacity of the adsorbent at *p* < *p*_tr_. Under these conditions, the statistical uncertainties associated with *Q*_m_ and *K* can be large even if the fit quality for the entire isotherm is good. For example, different values of *Q*_m_ and *K* obtained by fitting separately to the adsorption and desorption branches of an isotherm might simply reflect the larger number of data points measurable at *p* < $p_{tr}^{(ads)}$ during adsorption than at *p* < $p_{tr}^{(des)}$ during desorption.

Supplementary Table 1. Values and statistical standard uncertainties, *u*, of best fit parameters determined by regression of the LJMY-Langmuir model (eq (1) or Supplementary eq (3)) to the data of Mason et al. ^6^ for sorption of CH_4_ on Fe(bdp), together with the standard error of the fit.

| *T /* K | *p*_tr_/ MPa | u(*p*_tr_) / MPa | *σ* / MPa | u(*σ*) / MPa | *Q*_step_/ mol×kg^‑1^ | u(*Q*_step_) / mol·kg^‑1^ | *Q*_m_/ mol·kg^‑1^ | *K* / MPa^‑1^ | *γ* | u(*γ*) | Standard error / mol·kg^­-1^ |
| --- | --- | --- | --- | --- | --- | --- | --- | --- | --- | --- | --- |
| Adsorption | | | | | | | | | | | |
| 248 | 1.257 | 0.006 | 0.050 | 0.012 | 9.62 | 0.54 | 32.6 | 0.0520 |  |  | 0.54 |
| 261 | 1.615 | 0.008 | 0.066 | 0.014 | 8.82 | 0.45 | 359.4 | 0.0030 |  |  | 0.56 |
| 273 | 1.953 | 0.012 | 0.091 | 0.018 | 8.24 | 0.36 | 986.9 | 0.0010 |  |  | 0.55 |
| 285 | 2.337 | 0.014 | 0.116 | 0.021 | 7.98 | 0.37 | 1100.0 | 0.0010 |  |  | 0.57 |
| 298 | 2.715 | 0.017 | 0.160 | 0.026 | 8.10 | 0.40 | 1014.4 | 0.0010 |  |  | 0.59 |
| 311 | 3.170 | 0.025 | 0.261 | 0.039 | 8.44 | 0.48 | 1559.4 | 0.0004 |  |  | 0.62 |
| 323 | 3.493 | 0.018 | 0.275 | 0.028 | 8.01 | 0.32 | 850.7 | 0.0010 |  |  | 0.4 |
| Desorption | | | | | | | | | | | |
| 248 | 0.738 | 0.008 | 0.061 | 0.013 | 7.15 | 0.52 | 25.5 | 0.1190 |  |  | 0.5 |
| 261 | 0.904 | 0.008 | 0.072 | 0.014 | 6.47 | 0.50 | 30.7 | 0.0770 |  |  | 0.42 |
| 273 | 1.074 | 0.015 | 0.087 | 0.029 | 5.56 | 0.66 | 36.1 | 0.0590 |  |  | 0.45 |
| 285 | 1.258 | 0.017 | 0.130 | 0.029 | 5.83 | 0.64 | 177.1 | 0.0080 |  |  | 0.37 |
| 298 | 1.499 | 0.024 | 0.143 | 0.040 | 4.87 | 0.71 | 150.2 | 0.0090 |  |  | 0.31 |
| 311 | 1.671 | 0.027 | 0.183 | 0.049 | 4.60 | 0.52 | 1866.9 | 0.0005 |  |  | 0.29 |
| 323 | 1.865 | 0.064 | 0.230 | 0.100 | 3.90 | 1.20 | 2060.4 | 0.0002 |  |  | 0.35 |
| Adsorption and desorption branched fitted simultaneously | | | | | | | | | | | |
| 248 | 0.742 | 0.008 | 0.063 | 0.012 | 8.21 | 0.43 | 28.2 | 0.0829 | 0.701 | 0.023 | 0.58 |
| 261 | 0.907 | 0.009 | 0.080 | 0.013 | 7.94 | 0.40 | 84.9 | 0.0174 | 0.785 | 0.022 | 0.58 |
| 273 | 1.083 | 0.018 | 0.115 | 0.022 | 7.91 | 0.48 | 889.4 | 0.0012 | 0.807 | 0.034 | 0.63 |
| 285 | 1.278 | 0.020 | 0.143 | 0.023 | 7.72 | 0.42 | 1094.4 | 0.0009 | 0.831 | 0.031 | 0.60 |
| 298 | 1.508 | 0.034 | 0.182 | 0.037 | 7.43 | 0.65 | 1096.9 | 0.0007 | 0.804 | 0.042 | 0.67 |
| 311 | 1.726 | 0.042 | 0.231 | 0.039 | 7.12 | 0.43 | 1369.4 | 0.0005 | 0.833 | 0.045 | 0.65 |
| 323 | 1.944 | 0.052 | 0.266 | 0.049 | 6.91 | 0.55 | 978.8 | 0.0005 | 0.797 | 0.049 | 0.61 |

Supplementary Table 2. Values and statistical standard uncertainties, *u*, of best fit parameters determined by regression of the LJMY-Langmuir model (eq (1) ) or Supplementary eq (3)) to the data of Mason et al. ^6^ for sorption of CH_4_ on Co(bdp), together with the standard error of the fit.

| *T /* K | *p*_tr_/ MPa | u(*p*_tr_) / MPa | *σ* / MPa | u(*σ*) / MPa | *Q*_step_/ mol·kg^‑1^ | u(*Q*_step_) / mol·kg^‑1^ | *Q*_m_/ mol·kg^‑1^ | *K* /  MPa^‑1^ | *γ* | u(*γ*) | Standard error / mol·kg^­-1^ |
| --- | --- | --- | --- | --- | --- | --- | --- | --- | --- | --- | --- |
| Adsorption | | | | | | | | | | | |
| 273 | 1.317 | 0.009 | 0.095 | 0.016 | 6.73 | 0.31 | 284.3 | 0.0034 |  |  | 0.33 |
| 285 | 1.591 | 0.014 | 0.148 | 0.020 | 6.17 | 0.23 | 552.5 | 0.0015 |  |  | 0.35 |
| 298 | 1.906 | 0.021 | 0.192 | 0.033 | 5.58 | 0.33 | 916.3 | 0.0008 |  |  | 0.46 |
| 311 | 2.185 | 0.028 | 0.258 | 0.048 | 5.34 | 0.42 | 955.7 | 0.0007 |  |  | 0.48 |
| 323 | 2.443 | 0.031 | 0.336 | 0.058 | 5.64 | 0.52 | 995.7 | 0.0006 |  |  | 0.46 |
| Desorption | | | | | | | | | | | |
| 273 | 0.641 | 0.011 | 0.066 | 0.020 | 3.98 | 0.54 | 19.2 | 0.1374 |  |  | 0.4 |
| 285 | 0.745 | 0.018 | 0.087 | 0.033 | 3.33 | 0.62 | 19.7 | 0.1231 |  |  | 0.38 |
| 298 | 0.833 | 0.022 | 0.109 | 0.043 | 2.80 | 0.65 | 20.4 | 0.1101 |  |  | 0.34 |
| 311 | 0.894 | 0.031 | 0.102 | 0.061 | 1.77 | 0.63 | 23.4 | 0.0844 |  |  | 0.28 |
| 323 | 0.945 | 0.035 | 0.134 | 0.071 | 1.52 | 0.53 | 25.4 | 0.0700 |  |  | 0.22 |
| Adsorption and desorption branched fitted simultaneously | | | | | | | | | | | |
| 273 | 0.662 | 0.011 | 0.113 | 0.018 | 5.87 | 0.35 | 44.8 | 0.0291 | 1.00 | 0.04 | 0.44 |
| 285 | 0.780 | 0.017 | 0.163 | 0.025 | 5.51 | 0.35 | 401.3 | 0.0026 | 1.04 | 0.05 | 0.44 |
| 298 | 0.901 | 0.024 | 0.232 | 0.035 | 5.58 | 0.42 | 718.8 | 0.0011 | 1.12 | 0.06 | 0.48 |
| 311 | 1.024 | 0.038 | 0.318 | 0.055 | 5.24 | 0.50 | 993.2 | 0.0007 | 1.13 | 0.09 | 0.51 |
| 323 | 1.129 | 0.055 | 0.400 | 0.073 | 5.02 | 0.57 | 1018.2 | 0.0006 | 1.13 | 0.11 | 0.53 |

Supplementary Table 3. Values and statistical standard uncertainties, *u*, of best fit parameters determined by regression of the LJMY-Langmuir model (eq (1) ) or Supplementary eq (3)) to the data measured in this work (233 to 293 K) and by Arami-Niya et al.^7^ (303 K) for the sorption of CO_2_ on ZIF-7, together with the standard error of the fit.

| *T /* K | *p*_tr_/ MPa | u(*p*_tr_) / MPa | *σ* / MPa | u(*σ*) / MPa | *Q*_step_/ mol·kg^‑1^ | u(*Q*_step_) / mol·kg^‑1^ | *Q*_m_/ mol·kg^‑1^ | *K* / MPa^‑1^ | *γ* | u(*γ*) | Standard error / mol·kg^­-1^ |
| --- | --- | --- | --- | --- | --- | --- | --- | --- | --- | --- | --- |
| Adsorption | | | | | | | | | | | |
| 303 | 0.07925 | 0.00041 | 0.01303 | 0.00046 | 1.210 | 0.030 | 0.89 | 6.5 |  |  | 0.01 |
| 293 | 0.05183 | 0.00031 | 0.00960 | 0.00061 | 1.060 | 0.060 | 1.06 | 33.3 |  |  | 0.02 |
| 283 | 0.03128 | 0.00035 | 0.00547 | 0.00054 | 0.880 | 0.040 | 0.88 | 19.9 |  |  | 0.03 |
| 273 | 0.02305 | 0.00007 | 0.00395 | 0.00011 | 0.980 | 0.010 | 0.98 | 42.8 |  |  | 0.01 |
| 253 | 0.00767 | 0.00004 | 0.00354 | 0.00007 | 1.560 | 0.020 | 1.56 | 25.8 |  |  | 0.0005 |
| 247 | 0.00695 | 0.00001 | 0.00290 | 0.00002 | 1.510 | 0.010 | 1.51 | 37.1 |  |  | 0.001 |
| 244 | 0.00400 | 0.00110 | 0.00069 | 0.00104 | 0.930 | 0.360 | 0.93 | 132.2 |  |  | 0.01 |
| 238 | 0.00315 | 0.00003 | 0.00053 | 0.00010 | 1.290 | 0.050 | 1.29 | 157.8 |  |  | 0.01 |
| 233 | 0.00188 | 0.00000 | 0.00001 | 0.00000 | 1.310 | 0.030 | 1.31 | 272.3 |  |  | 0.01 |
| Desorption | | | | | | | | | | | |
| 303 | 0.04971 | 0.00118 | 0.00789 | 0.00200 | 1.200 | 0.030 | 0.87 | 7.5 |  |  | 0.01 |
| 293 | 0.03067 | 0.00016 | 0.00455 | 0.00023 | 0.870 | 0.020 | 0.87 | 21.4 |  |  | 0.01 |
| 283 | 0.01893 | 0.00007 | 0.00203 | 0.00012 | 0.880 | 0.020 | 0.88 | 36.0 |  |  | 0.01 |
| 273 | 0.01475 | 0.00000 | 0.00008 | 0.00000 | 0.870 | 0.020 | 0.87 | 69.2 |  |  | 0.01 |
| 253 | 0.00519 | 0.00000 | 0.00004 | 0.00000 | 0.920 | 0.020 | 0.92 | 192.5 |  |  | 0.01 |
| 247 | 0.00430 | 0.00006 | 0.00019 | 0.00009 | 1.360 | 0.080 | 1.36 | 76.4 |  |  | 0.02 |
| 244 | 0.00219 | 0.00001 | 0.00101 | 0.00002 | 1.290 | 0.010 | 1.29 | 160.5 |  |  | 0.001 |
| 238 | 0.00129 | 0.00012 | 0.00074 | 0.00017 | 1.530 | 0.320 | 1.53 | 95.8 |  |  | 0.09 |
| 233 | 0.00115 | 0.00015 | 0.00070 | 0.00021 | 1.660 | 0.330 | 1.66 | 22.6 |  |  | 0.14 |
| Adsorption and desorption branched fitted simultaneously | | | | | | | | | | | |
| 303 | 0.05120 | 0.00071 | 0.01160 | 0.00100 | 1.239 | 0.039 | 0.85 | 65.3 | 0.53 | 0.02 | 0.02 |
| 293 | 0.03031 | 0.00045 | 0.00712 | 0.00059 | 0.980 | 0.033 | 0.84 | 23.7 | 0.72 | 0.03 | 0.03 |
| 283 | 0.01870 | 0.00029 | 0.00311 | 0.00063 | 0.905 | 0.032 | 0.93 | 29.0 | 0.67 | 0.03 | 0.04 |
| 273 | 0.01293 | 0.00026 | 0.00274 | 0.00026 | 0.966 | 0.029 | 0.86 | 55.8 | 0.82 | 0.04 | 0.03 |
| 253 | 0.00421 | 0.00007 | 0.00105 | 0.00019 | 1.023 | 0.018 | 0.91 | 162.2 | 1.92 | 0.08 | 0.01 |
| 247 | 0.00353 | 0.00005 | 0.00001 | 0.00000 | 1.035 | 0.010 | 0.91 | 187.6 | 0.79 | 0.03 | 0.02 |
| 244 | 0.00218 | 0.00002 | 0.00104 | 0.00050 | 1.203 | 0.015 | 0.73 | 207.6 | 1.01 | 0.02 | 0.01 |
| 238 | 0.00128 | 0.00008 | 0.00071 | 0.00023 | 1.433 | 0.101 | 0.52 | 179.0 | 1.51 | 0.16 | 0.05 |
| 233 | 0.00117 | 0.00013 | 0.00066 | 0.00013 | 1.415 | 0.171 | 0.50 | 409.8 | 1.41 | 0.36 | 0.07 |

Supplementary Table 4. Values and statistical standard uncertainties, u, of best fit parameters determined by regression of the LJMY-Langmuir model (eq (1)) to the data of Couck et al. ^8^ for sorption of CO_2_ on CH_4_ on MIL-53(Al), together with the standard error of the fit.

| *T /* K | *p*_tr_/ MPa | u(*p*_tr_) / MPa | *σ* / MPa | u(*σ*) / MPa | *Q*_step_/ mol⋅kg^‑1^ | u(*Q*_step_) / mol⋅kg^‑1^ | *Q*_m_/ mol⋅kg^‑1^ | *K* / MPa^‑1^ | Standard error / mol⋅kg^­-1^ |
| --- | --- | --- | --- | --- | --- | --- | --- | --- | --- |
| CO_2_ – Adsorption | | | | | | | | | |
| 303 | 1.506 | 0.017 | 0.317 | 0.023 | 4.35 | 0.12 | 2.17 | 11.25 | 0.13 |
| 288 | 1.101 | 0.016 | 0.232 | 0.025 | 4.15 | 0.12 | 2.09 | 18.04 | 0.17 |
| CO_2_ – Desorption | | | | | | | | | |
| 303 | 0.606 | 0.049 | 0.312 | 0.054 | 4.86 | 0.52 | 1.08 | 31.59 | 0.21 |
| 288 | 0.406 | 0.015 | 0.08 | 0.02 | 3.88 | 0.31 | 2.19 | 28.40 | 0.24 |
| CH_4_ – Adsorption | | | | | | | | | |
| 303 | 1.028 | 0.01 | 0.149 | 0.015 | 0.71 | 0.03 | 5.37 | 0.17 | 0.02 |
| CH_4_ – Desorption | | | | | | | | | |
| 303 | 0.895 | 0.076 | 0.292 | 0.051 | 1.28 | 0.21 | 6.71 | 0.08 | 0.04 |

Regression of eq (2) to the values of *p*_tr_ measured for CO_2_ on MIL-53(Al) at 303 K and 288 K gives ${\Delta H}_{tr}^{(ads)}$= 15 kJ⋅mol^-1^ and ${\Delta H}_{tr}^{(des)}$= 19 kJ⋅mol^- 1^.


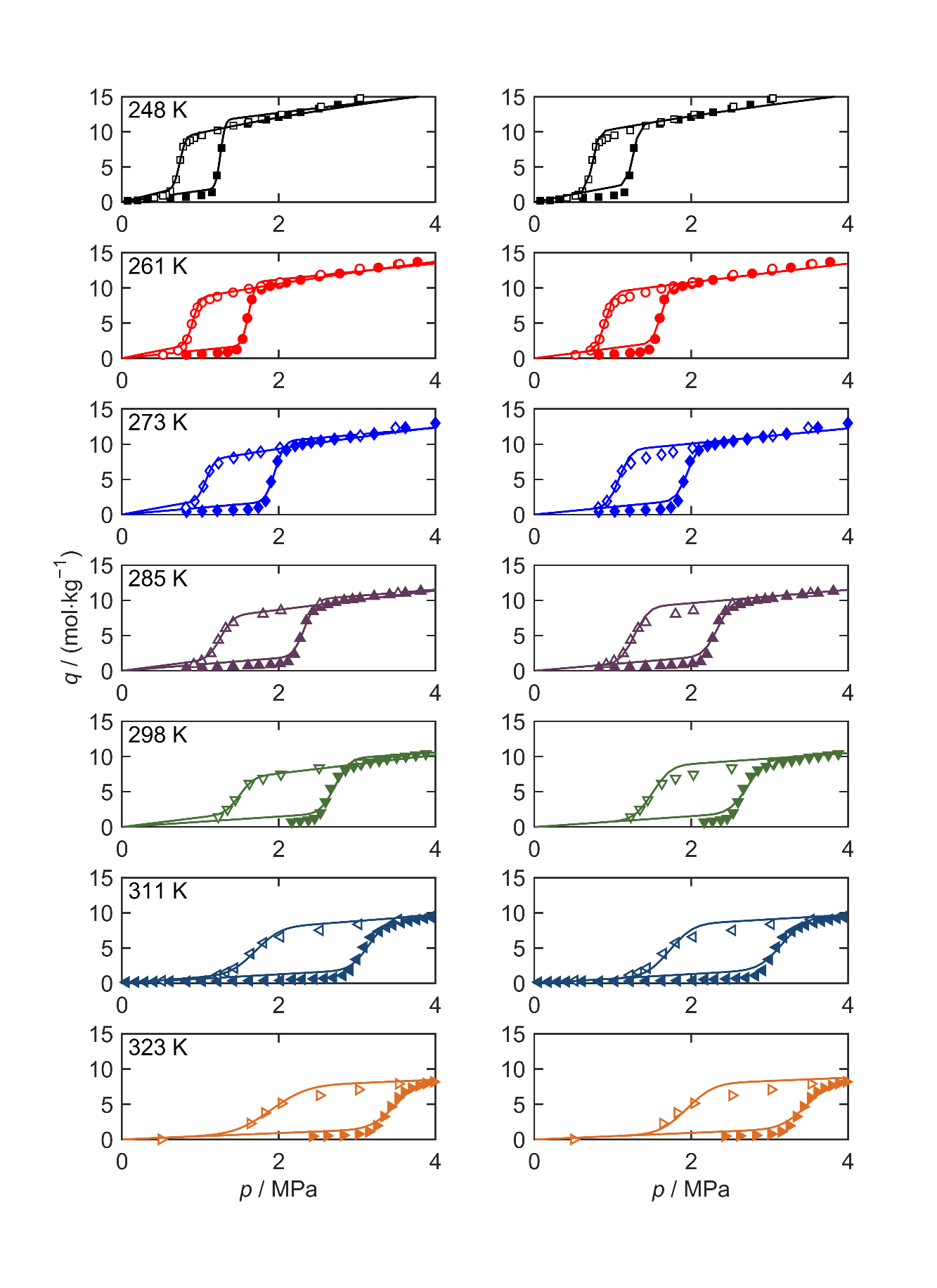


Supplementary Figure 1. Hysteretic sorption isotherms for CH_4_ on the Fe(bdp) measured by Mason et al.^9^ Symbols: experimental data; curves: calculated with LJMY-Langmuir model. (left) Adsorption or desorption branch at each temperature was fitted separately. (right) The adsorption and desorption branches at each temperature were fitted simultaneously.


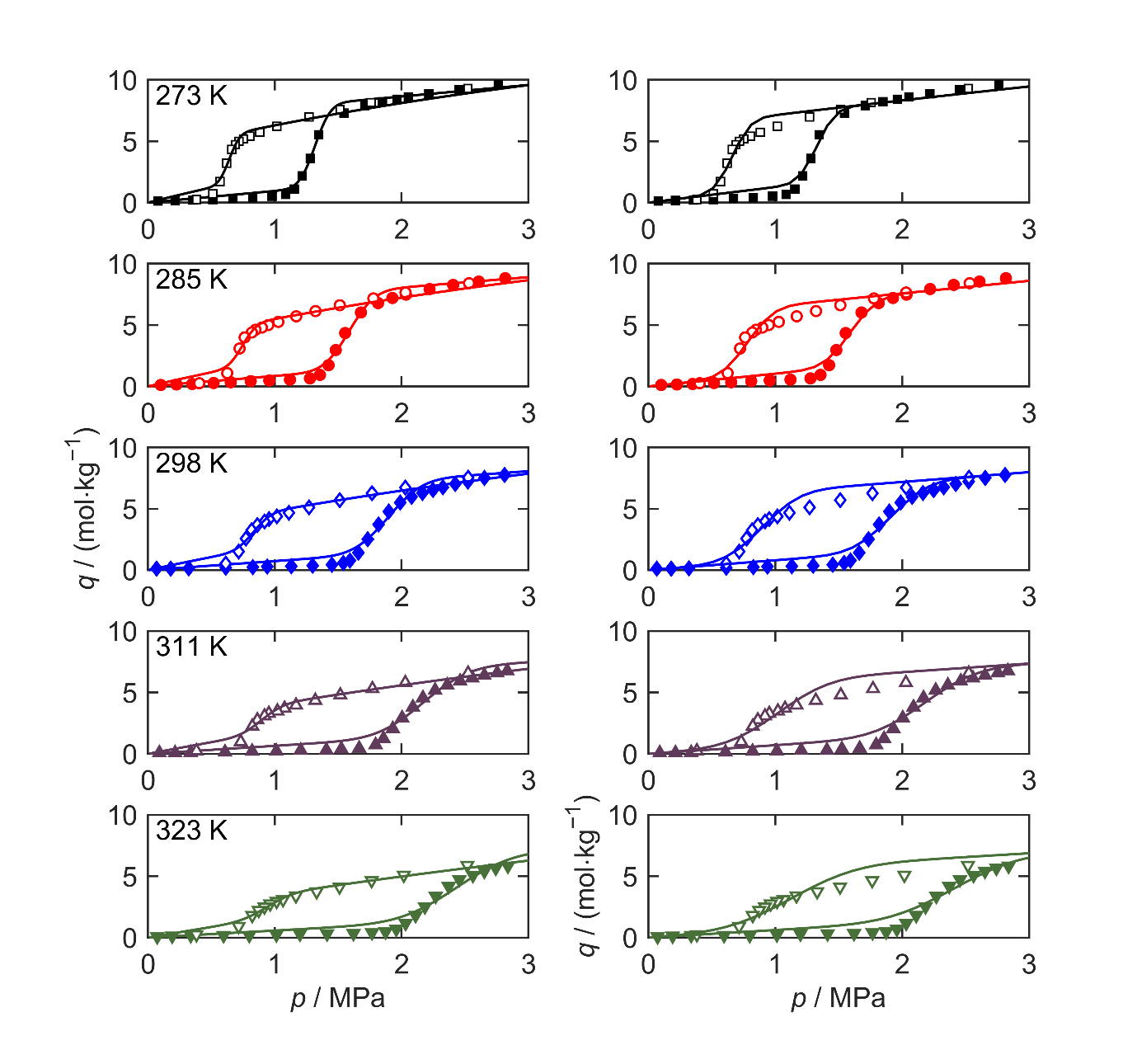


Supplementary Figure 2. Hysteretic sorption isotherms for CH_4_ on the Co(bdp) measured by Mason et al.^9^ Symbols: experimental data; curves: calculated with LJMY-Langmuir model. (left)Adsorption or desorption branch at each temperature was fitted separately. (right) The adsorption and desorption branches at each temperature were fitted simultaneously.


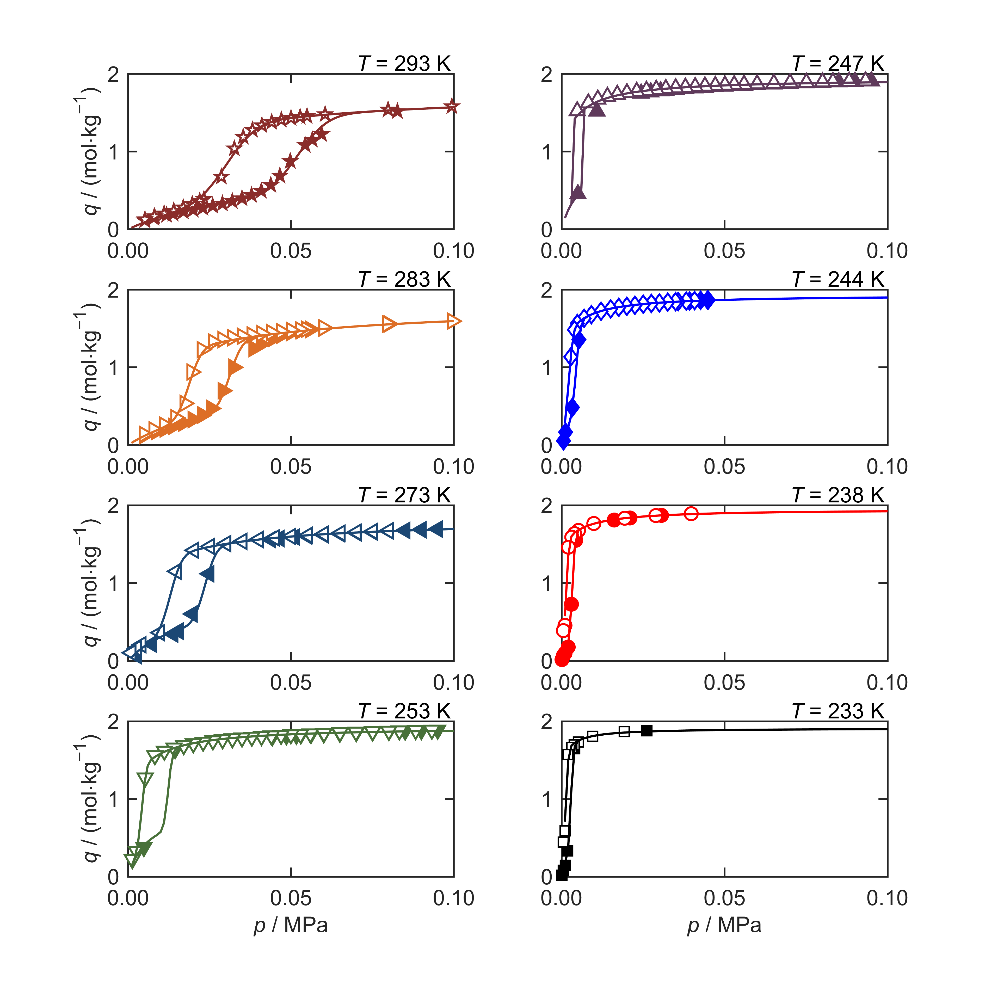


Supplementary Figure 3. Equilibrium CO_2_ capacities, *q*, measured in adsorption (filled symbols) and desorption (empty symbols) for ZIF-7. Curves represent fits of the LJMY‑Langmuir model with adsorption and desorption branched at each temperature fitted simultaneously.

## Supplementary Note 3. Measurements of CO_2_ Sorption on ZIF-7.

The ZIF-7 was synthesized following the method detailed by Arami Niya et al.^7^ A Micromeritics ASAP2020 instrument was used to measure the equilibrium capacities tabulated below:

Supplementary Table 5. Equilibrium capacities, *q*, of CO_2_ on ZIF-7 measured in adsorption and desorption as a function of pressure, *p*, and temperature, *T*. The relative combined standard uncertainty of sorption capacity, u_c_(q_i_)/q_i_, measured with this ASAP2020 was estimated previously ^10, 11^ to be 1.4 %.

| Adsorption | | |  | Desorption | | |
| --- | --- | --- | --- | --- | --- | --- |
| *T* / K | *p* / kPa | q / mol.kg^-1^ |  | *T* / K | *p* / kPa | q / mol.kg^-1^ |
| 233 | 0.094 | 0.020 |  | 233 | 19.210 | 1.868 |
| 233 | 0.463 | 0.084 |  | 233 | 9.539 | 1.808 |
| 233 | 1.134 | 0.147 |  | 233 | 5.116 | 1.735 |
| 233 | 1.867 | 0.333 |  | 233 | 3.872 | 1.696 |
| 233 | 3.965 | 1.646 |  | 233 | 3.119 | 1.663 |
| 233 | 26.087 | 1.879 |  | 233 | 1.912 | 1.573 |
|  |  |  |  | 233 | 1.038 | 0.591 |
|  |  |  |  | 233 | 0.491 | 0.445 |
| 238 | 0.096 | 0.017 |  | 238 | 39.755 | 1.893 |
| 238 | 0.479 | 0.064 |  | 238 | 28.915 | 1.869 |
| 238 | 1.028 | 0.098 |  | 238 | 19.337 | 1.835 |
| 238 | 1.997 | 0.178 |  | 238 | 9.868 | 1.767 |
| 238 | 2.998 | 0.729 |  | 238 | 5.175 | 1.679 |
| 238 | 4.300 | 1.551 |  | 238 | 3.994 | 1.637 |
| 238 | 16.122 | 1.812 |  | 238 | 3.102 | 1.592 |
| 238 | 20.958 | 1.838 |  | 238 | 2.082 | 1.463 |
| 238 | 30.621 | 1.872 |  | 238 | 1.017 | 0.457 |
| 238 | 39.755 | 1.893 |  | 238 | 0.511 | 0.390 |
| 244 | 0.558 | 0.055 |  | 244 | 40.803 | 1.859 |
| 244 | 1.232 | 0.163 |  | 244 | 38.209 | 1.853 |
| 244 | 3.319 | 0.483 |  | 244 | 35.721 | 1.847 |
| 244 | 5.304 | 1.356 |  | 244 | 34.969 | 1.845 |
| 244 | 39.251 | 1.853 |  | 244 | 32.530 | 1.839 |
| 244 | 42.612 | 1.862 |  | 244 | 29.975 | 1.831 |
| 244 | 44.745 | 1.867 |  | 244 | 27.494 | 1.822 |
| 244 | 44.951 | 1.868 |  | 244 | 25.008 | 1.812 |
|  |  |  |  | 244 | 22.490 | 1.800 |
|  |  |  |  | 244 | 20.024 | 1.787 |
|  |  |  |  | 244 | 17.508 | 1.771 |
|  |  |  |  | 244 | 15.046 | 1.752 |
|  |  |  |  | 244 | 12.100 | 1.722 |
|  |  |  |  | 244 | 9.142 | 1.679 |
|  |  |  |  | 244 | 6.890 | 1.632 |
|  |  |  |  | 244 | 4.797 | 1.564 |
|  |  |  |  | 244 | 3.918 | 1.484 |
|  |  |  |  | 244 | 2.791 | 1.134 |
| 247 | 4.974 | 0.454 |  | 247 | 100.039 | 1.913 |
| 247 | 10.819 | 1.519 |  | 247 | 93.244 | 1.908 |
| 247 | 24.376 | 1.750 |  | 247 | 88.228 | 1.904 |
| 247 | 27.335 | 1.766 |  | 247 | 83.229 | 1.899 |
| 247 | 30.383 | 1.780 |  | 247 | 80.034 | 1.896 |
| 247 | 31.882 | 1.787 |  | 247 | 75.074 | 1.890 |
| 247 | 35.129 | 1.800 |  | 247 | 70.081 | 1.884 |
| 247 | 38.003 | 1.809 |  | 247 | 65.106 | 1.878 |
| 247 | 40.932 | 1.818 |  | 247 | 59.964 | 1.871 |
| 247 | 44.045 | 1.827 |  | 247 | 55.965 | 1.865 |
| 247 | 46.949 | 1.834 |  | 247 | 52.933 | 1.860 |
| 247 | 50.009 | 1.841 |  | 247 | 50.102 | 1.855 |
| 247 | 53.055 | 1.847 |  | 247 | 47.128 | 1.849 |
| 247 | 56.070 | 1.853 |  | 247 | 43.960 | 1.842 |
| 247 | 60.043 | 1.861 |  | 247 | 40.955 | 1.835 |
| 247 | 65.084 | 1.869 |  | 247 | 37.966 | 1.828 |
| 247 | 69.974 | 1.876 |  | 247 | 34.983 | 1.819 |
| 247 | 75.050 | 1.883 |  | 247 | 31.953 | 1.810 |
| 247 | 80.019 | 1.890 |  | 247 | 29.014 | 1.800 |
| 247 | 85.041 | 1.896 |  | 247 | 26.007 | 1.787 |
| 247 | 89.999 | 1.902 |  | 247 | 23.014 | 1.773 |
| 247 | 95.047 | 1.908 |  | 247 | 20.021 | 1.756 |
| 247 | 100.039 | 1.913 |  | 247 | 17.062 | 1.735 |
|  |  |  |  | 247 | 14.035 | 1.709 |
|  |  |  |  | 247 | 11.091 | 1.674 |
|  |  |  |  | 247 | 8.179 | 1.625 |
|  |  |  |  | 247 | 4.770 | 1.524 |
| 253 | 4.815 | 0.378 |  | 253 | 100.030 | 1.895 |
| 253 | 14.433 | 1.639 |  | 253 | 93.259 | 1.889 |
| 253 | 44.292 | 1.805 |  | 253 | 88.280 | 1.884 |
| 253 | 48.597 | 1.817 |  | 253 | 83.272 | 1.879 |
| 253 | 51.649 | 1.824 |  | 253 | 80.031 | 1.876 |
| 253 | 53.014 | 1.828 |  | 253 | 75.071 | 1.870 |
| 253 | 56.135 | 1.834 |  | 253 | 69.952 | 1.863 |
| 253 | 60.061 | 1.841 |  | 253 | 65.050 | 1.856 |
| 253 | 65.001 | 1.850 |  | 253 | 60.116 | 1.848 |
| 253 | 70.046 | 1.858 |  | 253 | 55.948 | 1.841 |
| 253 | 75.052 | 1.865 |  | 253 | 52.936 | 1.835 |
| 253 | 80.067 | 1.872 |  | 253 | 49.967 | 1.829 |
| 253 | 85.052 | 1.878 |  | 253 | 46.957 | 1.822 |
| 253 | 90.027 | 1.884 |  | 253 | 43.974 | 1.815 |
| 253 | 95.052 | 1.889 |  | 253 | 40.949 | 1.807 |
| 253 | 100.030 | 1.895 |  | 253 | 37.978 | 1.798 |
|  |  |  |  | 253 | 34.949 | 1.789 |
|  |  |  |  | 253 | 32.008 | 1.778 |
|  |  |  |  | 253 | 28.987 | 1.765 |
|  |  |  |  | 253 | 26.027 | 1.751 |
|  |  |  |  | 253 | 23.014 | 1.734 |
|  |  |  |  | 253 | 20.008 | 1.714 |
|  |  |  |  | 253 | 17.054 | 1.690 |
|  |  |  |  | 253 | 14.065 | 1.658 |
|  |  |  |  | 253 | 11.106 | 1.618 |
|  |  |  |  | 253 | 8.206 | 1.560 |
|  |  |  |  | 253 | 5.227 | 1.273 |
|  |  |  |  | 253 | 2.576 | 0.323 |
|  |  |  |  | 253 | 1.278 | 0.222 |
| 273 | 2.617 | 0.067 |  | 273 | 100.014 | 1.703 |
| 273 | 7.219 | 0.217 |  | 273 | 93.423 | 1.695 |
| 273 | 13.795 | 0.345 |  | 273 | 88.327 | 1.688 |
| 273 | 15.425 | 0.383 |  | 273 | 83.368 | 1.680 |
| 273 | 19.720 | 0.604 |  | 273 | 79.937 | 1.674 |
| 273 | 24.830 | 1.121 |  | 273 | 75.120 | 1.665 |
| 273 | 43.801 | 1.561 |  | 273 | 70.046 | 1.655 |
| 273 | 46.423 | 1.574 |  | 273 | 65.139 | 1.643 |
| 273 | 51.191 | 1.593 |  | 273 | 60.011 | 1.630 |
| 273 | 54.956 | 1.606 |  | 273 | 55.162 | 1.617 |
| 273 | 60.003 | 1.622 |  | 273 | 49.984 | 1.601 |
| 273 | 65.044 | 1.636 |  | 273 | 44.970 | 1.583 |
| 273 | 70.072 | 1.648 |  | 273 | 39.990 | 1.562 |
| 273 | 75.042 | 1.659 |  | 273 | 35.004 | 1.537 |
| 273 | 80.049 | 1.669 |  | 273 | 30.047 | 1.508 |
| 273 | 85.036 | 1.679 |  | 273 | 25.073 | 1.472 |
| 273 | 90.052 | 1.687 |  | 273 | 20.207 | 1.423 |
| 273 | 95.026 | 1.696 |  | 273 | 14.809 | 1.153 |
| 273 | 100.014 | 1.703 |  | 273 | 9.719 | 0.363 |
|  |  |  |  | 273 | 3.995 | 0.208 |
|  |  |  |  | 273 | 2.581 | 0.163 |
|  |  |  |  | 273 | 0.919 | 0.105 |
| 283 | 4.887 | 0.119 |  | 283 | 99.522 | 1.595 |
| 283 | 8.593 | 0.173 |  | 283 | 79.641 | 1.559 |
| 283 | 11.262 | 0.206 |  | 283 | 60.039 | 1.508 |
| 283 | 13.821 | 0.239 |  | 283 | 54.698 | 1.491 |
| 283 | 16.983 | 0.282 |  | 283 | 52.825 | 1.484 |
| 283 | 19.874 | 0.328 |  | 283 | 50.112 | 1.474 |
| 283 | 22.848 | 0.386 |  | 283 | 46.964 | 1.461 |
| 283 | 25.602 | 0.470 |  | 283 | 43.971 | 1.448 |
| 283 | 29.106 | 0.699 |  | 283 | 40.970 | 1.434 |
| 283 | 32.381 | 0.999 |  | 283 | 37.976 | 1.418 |
| 283 | 38.553 | 1.243 |  | 283 | 34.985 | 1.401 |
| 283 | 40.825 | 1.296 |  | 283 | 32.013 | 1.381 |
| 283 | 44.605 | 1.373 |  | 283 | 29.029 | 1.358 |
| 283 | 46.934 | 1.410 |  | 283 | 26.131 | 1.326 |
| 283 | 50.024 | 1.447 |  | 283 | 22.671 | 1.228 |
| 283 | 53.027 | 1.471 |  | 283 | 19.514 | 0.940 |
| 283 | 55.990 | 1.488 |  | 283 | 17.398 | 0.535 |
| 283 | 59.988 | 1.504 |  | 283 | 14.576 | 0.351 |
| 283 | 80.031 | 1.558 |  | 283 | 11.034 | 0.258 |
| 283 | 99.522 | 1.595 |  | 283 | 7.865 | 0.195 |
|  |  |  |  | 283 | 4.868 | 0.137 |
| 293 | 5.084 | 0.113 |  | 293 | 99.399 | 1.582 |
| 293 | 7.853 | 0.145 |  | 293 | 79.814 | 1.540 |
| 293 | 11.803 | 0.182 |  | 293 | 60.472 | 1.480 |
| 293 | 13.776 | 0.199 |  | 293 | 54.912 | 1.456 |
| 293 | 16.960 | 0.225 |  | 293 | 52.791 | 1.446 |
| 293 | 19.961 | 0.249 |  | 293 | 50.005 | 1.430 |
| 293 | 22.931 | 0.274 |  | 293 | 47.033 | 1.409 |
| 293 | 25.957 | 0.300 |  | 293 | 44.076 | 1.379 |
| 293 | 28.942 | 0.329 |  | 293 | 41.068 | 1.337 |
| 293 | 31.968 | 0.361 |  | 293 | 38.154 | 1.279 |
| 293 | 34.945 | 0.396 |  | 293 | 35.325 | 1.189 |
| 293 | 37.904 | 0.438 |  | 293 | 32.593 | 1.039 |
| 293 | 40.890 | 0.493 |  | 293 | 28.611 | 0.675 |
| 293 | 43.795 | 0.568 |  | 293 | 23.254 | 0.387 |
| 293 | 46.576 | 0.689 |  | 293 | 21.973 | 0.364 |
| 293 | 49.771 | 0.876 |  | 293 | 19.693 | 0.324 |
| 293 | 54.347 | 1.084 |  | 293 | 16.907 | 0.279 |
| 293 | 56.536 | 1.151 |  | 293 | 13.998 | 0.238 |
| 293 | 59.389 | 1.223 |  | 293 | 11.034 | 0.201 |
| 293 | 82.688 | 1.519 |  | 293 | 8.116 | 0.166 |
| 293 | 99.399 | 1.582 |  | 293 | 5.154 | 0.126 |

## Supplementary Note 4. Temperature dependence and hysteresis of structural transition parameters.

In the absence of structural information about a given adsorbent, observations regarding the temperature dependence of any hysteresis in the sorption isotherm could help identify the nature of the material. For example, the degree of isotherm hysteresis increases with decreasing temperature when the hysteresis is caused by capillary condensation in the mesopores of a rigid material. This is in contrast to the increase in isotherm hysteresis with increasing temperature associated flexible adsorbent structural transformations. Potentially, investigating the temperature dependence and resulting hysteresis of the isotherm’s transition pressures and/or transition widths could provide additional insight into the nature of the material’s structural transformation.

For example, from eq (2) if the ratio ${p_{tr}^{(ads)}}/{p_{{tr}_{0}}^{(ads)}}$ changes more with increasing temperature than ${p_{tr}^{(des)}}/{p_{{tr}_{0}}^{(des)}}$ , then ${\Delta H}_{tr}^{(ads)}$ is larger than ${\Delta H}_{tr}^{(des)}$. For CO_2_ on ZIF-7 over the temperature range considered in this work, these enthalpy changes for each branch are statistically equivalent. For CH_4_ on Co(bdp), ${\Delta H}_{tr}^{(ads)}$ are 60 % larger than ${\Delta H}_{tr}^{(des)}$, while for CO_2_ on MIL-53(Al), ${\Delta H}_{tr}^{(ads)}$ is 22 % smaller than ${\Delta H}_{tr}^{(des)}$. Further research is needed to investigate how these differences in ${\delta H}_{tr}\equiv{\Delta H}_{tr}^{(ads)}-{\Delta H}_{tr}^{(des)}$ correspond to the specific nature of the structural transformation.

Such information may also be encoded within the transition width’s temperature dependence and hysteresis. Supplementary Figure 4 shows the variation in this parameter for both branches of the sorption isotherms measured for CH_4_ on Fe(bdp), CH_4_ on Co(bdp) and CO_2_ on ZIF-7. No hysteresis in the transition width is resolvable for CH_4_ on Fe(bdp) while for Co(bdp) the difference in σ between the two branches is clear and increases with temperature. For CO_2_ on ZIF-7 below 247 K the values of σ determined for the adsorption and desorption branches are essentially statistically equivalent: at higher temperatures a sustained difference in the range (3 to 5) kPa is observed. Collectively, the results from these three MOFs suggest there might be a temperature below which σ is the same for both branches of the isotherm, while at higher temperatures a hysteresis in this parameter becomes increasingly manifest. As suggested in Supplementary Section 1, differences in the value of σ obtained for each branch might reflect differences in the crystal strain caused by the direction of the **np-lp** transition. Alternatively (or in addition) the magnitude and temperature dependence of σ might contain information about the sample’s heterogeneity. Clearly, however, such speculation needs to be investigated further by measuring hysteretic sorption isotherms over a wider range of *T*, with additional MOF-adsorbate combinations, and through fundamental simulations with methods such as those of Evans et al.^3^

Supplementary Figure 4. Temperature dependence and hysteresis of the transition width parameter, *σ*, reported in Supplementary Tables 1 to 3 when each branch is fit separately to eq (1).

## Supplementary Note 5. Pressure Vacuum Swing Adsorption simulation of a CH_4_/CO_2_ mixture with ZIF‑7.

In principle, MOFs have significant potential for use in gas separation processes as a result of their characteristic stepped isotherms, which could deliver benefits in terms of both selectivity and working capacity. However, studies of process applications involving MOFs remain comparatively limited in part because of the difficulty associated with the description of the stepped and hysteretic sorption isotherms within the frameworks provided by process simulators. Here we demonstrate the use of the LJMY-Langmuir isotherm model within a simulation of a Pressure Vacuum Swing Adsorption (PVSA) process separating an equimolar mixture of methane + carbon dioxide using ZIF-7. The study reveals the importance of rigorously considering the sorption isotherms, and particularly their hysteresis, in both the design and simulation of the separation process.

A two-bed, four-step PVSA process was simulated numerically using the software package Aspen Adsorption. A graphical layout of the simulation is shown in Supplementary Figure 5, and the step sequence is listed in Supplementary Table 6.


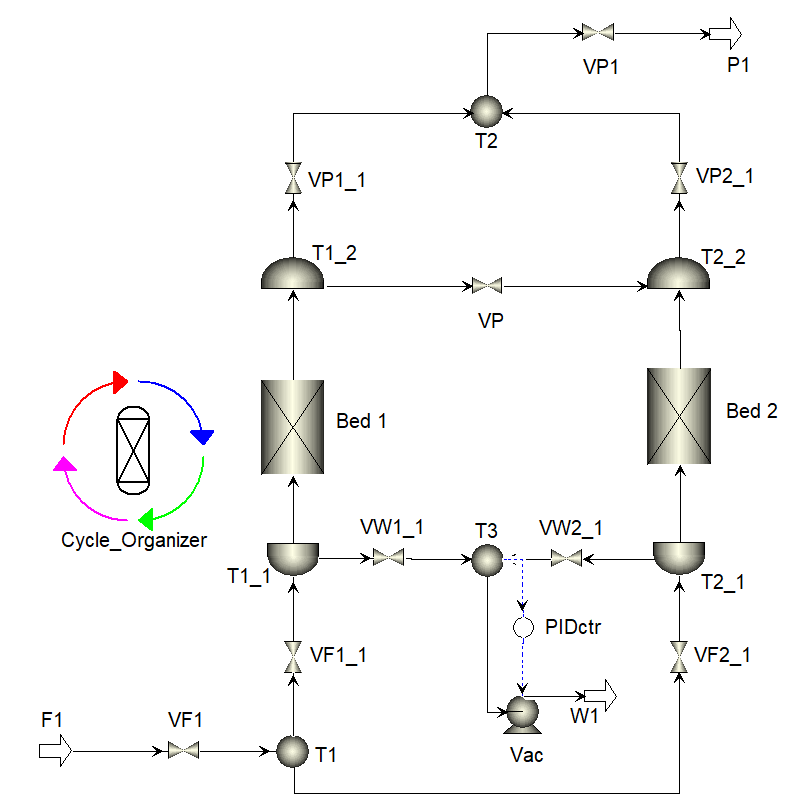


Supplementary Figure 5. Graphical layout of the PVSA model built with Aspen Adsorption.

Supplementary Table 6. PVSA cycle step sequence and timing

|  | Step 1 | Step 2 | Step 3 | Step 4 |
| --- | --- | --- | --- | --- |
| Time (s) | 60 | 60 | 60 | 60 |
| Bed 1 | Adsorption | Blowdown | Evacuate | Repressurisation |
| Bed 2 | Evacuate | Repressurisation | Adsorption | Blowdown |

The column dimensions, adsorbent properties, and process conditions are listed in Supplementary Table 7, while the isotherm parameters for CO_2_ and CH_4_ on ZIF-7 at 303 K are listed in Supplementary Table 8. The values of the ZIF-7 isotherm parameters were obtained by fits of eq (1) to the data measured by Arami-Niya et al.^7^ for CO_2_ and to the data reported recently by Yang et al.^12^ for CH_4_. The isotherm data and corresponding fits of the LJMY-Langmuir models are shown in Supplementary Figure 6.

Supplementary Table 7. Simulation parameters

| **Parameter** | **Value** |
| --- | --- |
| Nodes per column | 20 |
| Height of column (m) | 0.3 |
| Diameter of column (m) | 0.03 |
| Inter-particle void fraction | 0.348 |
| Intra-particle void fraction | 0.146 |
| Bulk solid density (kg/m^3^) | 385.0 |
| Particle radius (m) | 0.0007 |
| Mass Transfer Coefficient – CH_4_ (s^-1^) | 1 |
| Mass Transfer Coefficient – CO_2_ (s^-1^) | 1 |
| Column and gas temperature (K) | 303.15 |
| p_high_ in column (bar) | 2, 1.5 |
| p_low_ in column (bar) | 1.0, 0.8, or 0.6 |
| Feed flowrate (mol/s) | 3.4 × 10^-3^ |
| Feed gas mole fraction composition | 0.5 CH_4_ + 0.5 CO_2_ |

Supplementary Table 8. Values of the LJMY-Langmuir sorption isotherm parameters for ZIF-7 at 303 K used to simulate a PVSA separation of an equimolar CO_2_ + CH_4_ mixture, based on the data reported by Aram-Niya et al.^7^ and by Yang et al. ^12^.

| Parameter | CO_2_ | | CH_4_ | |
| --- | --- | --- | --- | --- |
|  | Adsorption | Desorption | Adsorption | Desorption |
| *p*_tr_ / bar | 0.793 | 0.497 | 6.99 | 4.76 |
| *σ* / bar | 0.130 | 0.079 | 0.73 | 0.61 |
| *Q*_step_ / mol⋅kg^-1^ | 1.20 | 1.20 | 1.00 | 1.00 |
| *K* / mol⋅kg^-1^⋅bar^-1^ | 0.65 | 0.75 | 0.20 | 0.32 |
| *Q*_m_ / mol⋅kg^-1^ | 0.89 | 0.89 | 0.88 | 0.79 |


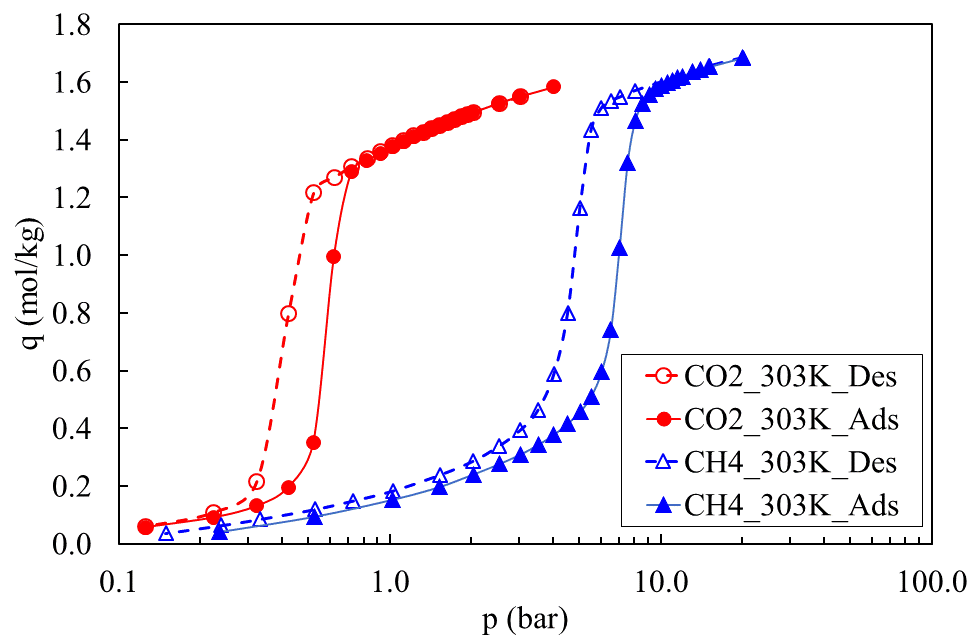


Supplementary Figure 6. Isotherm data and LJMY-Langmuir fits for CO_2_ and CH_4_ on ZIF‑7 at 303 K.

A user-defined isotherm model was implemented in Aspen Adsorption for both beds. The simulation could be run using either (i) only the adsorption branch, (ii) only the desorption branch, or (iii) both branches as part of a fully hysteretic model. In this third case, for a given step of the PVSA cycle the appropriate branch of the isotherm was selected via a programming flag: during the “Adsorption” and “Repressurisation” steps, the adsorption branch of the isotherm was selected, while for the “Blowdown” and “Evacuate” steps, the desorption branch was selected. Sorption was assumed to be non-competitive: the equilibrium capacity of each component at a given node within the bed was evaluated from the partial pressure of that component in the gas phase at that node. In principle, competitive adsorption effects could be simulated using, for example, the Osmotic Framework Adsorbed Solution Theory proposed by Coudert et al. ^2^.

To ensure complete utilisation of the step in the CO_2_ isotherm by the PVSA process, the CO_2_ *partial* pressure needs to swing from $p_{high}>p_{tr}^{(ads)}+\sigma^{(\mathrm{ads})}$ to $p_{low}<p_{tr}^{(des)}-\sigma^{(\mathrm{des})}$. The selectivity inferred from the pure fluid isotherms reaches a maximum of about 7 at a (partial) pressure of $p_{tr}^{(ads)}+\sigma^{(\mathrm{ads})}$; at higher pressures the selectivity decreases as the CH_4_ uptake increases faster than the CO_2_ uptake. To investigate the impact of varying the pressure swing limits in the PVSA cycle on the separation performance, simulations were conducted for a high pressure of (2 and 1.5) bar, with desorption pressures of either (1, 0.8, 0.6 or 0.4) bar; for an equimolar mixture, these values span the partial pressure limits identified above. The methane mole fraction of the light product stream (P1 in Supplementary Figure 5) was the metric used to assess changes in separation performance. These pressure swing limits and the resulting separation performance were also analysed in the context of the model used to represent the CO_2_ sorption isotherm: either fully hysteretic, just the adsorption branch or just the desorption branch. Supplementary Figure 7 shows the light product methane mole fraction produced by a PVSA cycle using ZIF-7 with *p*_high_ = 2 bar at the four values of *p*_low_ considered, when the simulation used each of the three isotherm models.

Supplementary Figure 7. Simulated methane content of light product (P1 stream in Supplementary Figure 5) produced from an equimolar feed of CH_4_ + CO_2_ using ZIF-7 in a 2-bed, 4-step PVSA process with *p*_high_= 2 bar. For every desorption pressure (*p*_low_) considered the result of using each of the three isotherm models (adsorption branch, desorption branch or full hysteresis) is shown.

There are three key features of the results shown in Supplementary Figure 7 that inform the design and simulation of PVSA cycles using a flexible MOF like ZIF-7 with a hysteretic stepped isotherm.

1. The separation performance predicted using the fully hysteretic isotherm model is typically worse than the performance predicted using either branch alone.
2. Single-branch isotherm models will predict *significantly* better separation performance than the fully hysteretic model *if* the pressure swing limits *p*_high_ or *p*_low_ are not both clear of the partial pressure limits $p_{tr}^{(ads)}+\sigma^{(\mathrm{ads})}$ or $p_{tr}^{(des)}-\sigma^{(\mathrm{des})}$, respectively.
3. If the pressure swing limits are consistent with the partial pressure limits required for a good separation, the performance predicted using the branch with the smallest transition width, *σ*, will be the more (overly) optimistic.

There are two primary reasons for these features. The most important is the need for the pressure swing limits to be beyond the partial pressure limits set by the locations and widths of the steps on the two branches. The parameters in the LJMY isotherm model allow these to be readily identified, so that *p*_high_ and *p*_low_ can be chosen to take advantage of the full isotherm step. For example, if only the adsorption branch is considered in the simulation and *p*_low_ is chosen so that the partial pressure is below $p_{tr}^{(ads)}$ but not below $p_{tr}^{(des)}-\sigma^{(\mathrm{des})}$ then an overly optimistic separation performance will be predicted. This explains the high adsorption branch result observed for *p*_low_ = (1 and 0.8) bar in Supplementary Figure 7. The converse also applies: if *p*_high_ is chosen so that the partial pressure is above $p_{tr}^{(des)}$ but not above $p_{tr}^{(ads)}+\sigma^{(\mathrm{ads})}$, then simulations based only on the desorption branch isotherm will predict significantly better separation performance than those based on either the fully hysteretic model or the adsorption branch model. The simulations conducted with *p*_high_ = 1.5 bar in this work produce results with a similar pattern to that shown in Supplementary Figure 7, except that light product methane fractions are lower for all cases, reflecting the reduction in selectivity during the adsorption step. However, when *p*_low_ was (0.6 or 0.4) bar, the desorption branch model predicted CH_4_ fractions around 0.8, similar to those shown in Supplementary Figure 7. In contrast, the fully hysteretic and adsorption branch models predicted CH_4_ fractions around 0.67 because *p*_high_ was not sufficient to clear the upper partial pressure limit of $p_{tr}^{(ads)}+\sigma^{(\mathrm{ads})}$.

The second reason for the three features identified is that sharper isotherm steps produce better separation performance. During the adsorption stage of the PVSA cycle, the partial pressure of the adsorbate varies spatially across the bed according to the gas-phase composition profile. Narrower steps in the adsorption isotherm will act to sharpen the gas composition front in the bed and improve the separation performance. For ZIF-7, $\sigma^{(\mathrm{des})}<\sigma^{(\mathrm{ads})}$ so models based on the desorption branch will always predict better performance than those based on the adsorption branch, provided the pressure swing limits are appropriate. Imposing two different widths in the fully hysteretic model typically results in a separation performance that is slightly worse than that predicted using the adsorption branch model.

## Supplementary References

1. Schneemann, A.; Bon, V.; Schwedler, I.; Senkovska, I.; Kaskel, S.; Fischer, R. A., Flexible metal-organic frameworks. *Chemical Society Reviews* **2014,** *43* (16), 6062-6096.

2. Coudert, F.-X.; Jeffroy, M.; Fuchs, A. H.; Boutin, A.; Mellot-Draznieks, C., Thermodynamics of Guest-Induced Structural Transitions in Hybrid Organic−Inorganic Frameworks. *Journal of the American Chemical Society* **2008,** *130* (43), 14294-14302.

3. Evans, J. D.; Krause, S.; Kaskel, S.; Sweatman, M. B.; Sarkisov, L., Exploring the thermodynamic criteria for responsive adsorption processes. *Chemical Science* **2019,** *10* (19), 5011-5017.

4. Krause, S.; Evans, J. D.; Bon, V.; Senkovska, I.; Coudert, F.-X.; Többens, D. M.; Wallacher, D.; Grimm, N.; Kaskel, S., The Role of Temperature and Adsorbate on Negative Gas Adsorption in the Mesoporous Metal-Organic Framework DUT-49. *Faraday Discussions* **2020**.

5. Neimark, A. V.; Coudert, F.-X.; Boutin, A.; Fuchs, A. H., Stress-Based Model for the Breathing of Metal−Organic Frameworks. *The Journal of Physical Chemistry Letters* **2010,** *1* (1), 445-449.

6. Mason, J. A.; Oktawiec, J.; Taylor, M. K.; Hudson, M. R.; Rodriguez, J.; Bachman, J. E.; Gonzalez, M. I.; Cervellino, A.; Guagliardi, A.; Brown, C. M.; Llewellyn, P. L.; Masciocchi, N.; Long, J. R., Methane storage in flexible metal–organic frameworks with intrinsic thermal management. *Nature* **2015,** *527* (7578), 357-361.

7. Arami-Niya, A.; Birkett, G.; Zhu, Z.; Rufford, T. E., Gate opening effect of zeolitic imidazolate framework ZIF-7 for adsorption of CH_4_ and CO_2_ from N_2_. *J. Mater. Chem. A* **2017,** *5* (40), 21389-21399.

8. Couck, S.; Denayer, J. F. M.; Baron, G. V.; Rémy, T.; Gascon, J.; Kapteijn, F., An Amine-Functionalized MIL-53 Metal−Organic Framework with Large Separation Power for CO2 and CH4. *Journal of the American Chemical Society* **2009,** *131* (18), 6326-6327.

9. Mason, J. A.; Oktawiec, J.; Taylor, M. K.; Hudson, M. R.; Rodriguez, J.; Bachman, J. E.; Gonzalez, M. I.; Cervellino, A.; Guagliardi, A.; Brown, C. M., Methane storage in flexible metal–organic frameworks with intrinsic thermal management. *Nature* **2015,** *527* (7578), 357-361.

10. Wedler, C.; Arami-Niya, A.; Xiao, G.; Span, R.; May, E. F.; Richter, M., Gas Diffusion and Sorption in Carbon Conversion. *Energy Procedia* **2019,** *158*, 1792-1797.

11. Xiao, G.; Li, Z.; Saleman, T. L.; May, E. F., Adsorption equilibria and kinetics of CH4 and N2 on commercial zeolites and carbons. *Adsorption* **2017,** *23* (1), 131-147.

12. Yang, X.; Arami-Niya, A.; Xiao, G.; May, E., Flexible Adsorbents at High Pressure: Observations and Correlation of ZIF-7 Sorption Isotherms for Argon, Nitrogen and Other Gases. *Langmuir* **2020,** *Submitted*.
